# A Higher Order Iterative Method for Computing the Drazin Inverse

**DOI:** 10.1155/2013/708647

**Published:** 2013-09-30

**Authors:** F. Soleymani, Predrag S. Stanimirović

**Affiliations:** ^1^Department of Mathematics, Zahedan Branch, Islamic Azad University, Zahedan, Iran; ^2^Faculty of Sciences and Mathematics, University of Niš, Višegradska 33, 18000 Niš, Serbia

## Abstract

A method with high convergence rate for finding approximate inverses of nonsingular matrices is suggested and established analytically. An extension of the introduced computational scheme to general square matrices is defined. The extended method could be used for finding the Drazin inverse. The application of the scheme on large sparse test matrices alongside the use in preconditioning of linear system of equations will be presented to clarify the contribution of the paper.

## 1. Introduction

 Computing the matrix inverse of nonsingular matrices of higher sizes is difficult and is a time consuming task. Application of higher order algorithms to solve this problem is very desirable. Generally speaking, in wide variety of topics, one must compute the inverse or particularly the generalized inverses to comprehend and realize significant features of the involved problems [1]. An example could be in phased-array radar whereas the target tracking is a recursive prediction correction process, when Kalman filtering is extensively consumed; see [2, 3]. Target equations are modeled explicitly such that the position and velocity and potentially higher derivatives of each measurement are approximated by the track filter as a state vector. The approximated error with the state vector is modeled by taking into account a covariance matrix, which is then used in subsequent computations. To be more precise, this matrix gets updated in each iteration of the track filter. Finding the inverse in the next iteration could make use of the inverse in the present iteration. In this circumstance, fast and efficient iterative algorithms are required.

There are some techniques to tackle this problem, which are basically divided into two main parts: the direct solvers such as Gaussian Elimination with Partial Pivoting (GEPP), which requires a massive load of computations and memory for large scale problems, and the iterative methods of the class of Schulz-type iterations, in which an approximation of the matrix inverse (by using a threshold) can be found per step up to the desired accuracy.

Almost all of the direct methods for matrix inversion require high accuracy in the computations to attain proper solutions as they are not tolerant to errors in the computed matrices. In contrast, iterative method compensates for individual and accumulation of round-off errors as it is a process of successive refinement.

In this paper, we focus on presenting and demonstrating a new iterative method to find approximate inverse matrices as fast as possible with a close attention in reducing the computational time. Toward this goal, a theoretical discussion will also be given to show the behavior of the proposed scheme. An interesting point in the contribution is that it could easily be applied to complex matrices as well as for finding the Drazin inverse.

To clarify the procedure, we now remind of some of the well-known methods in what follows. Perhaps, the most common technique to compute the inverse of a nonsingular complex matrix *A*, is the Schulz method given in [4] as follows:

(1) $$\begin{array}{c} V_{n+1}=V_{n}\left(2I-AV_{n}\right),\;\;n=0,1,2,\ldots , \end{array}$$

where *I* is the identity matrix with the same dimension that of the matrix *A*. The scheme (1) has become popular in the 1980s due to the emerging of parallel machines.

Such iterative methods are sensitive for the initial guess/value (*V*_0_) to start the process and converge to *A* ^−1^. In practice, the Schulz-type methods are efficient (specially for structured matrices) but a difficulty lies in the initial approximation of the *A* ^−1^. This need was fulfilled by providing some appropriate initial approximations in the literature. For example, Rajagopalan in [5] gave some initial approximations for the inverse matrix by considering different norms as:

(2) $$\begin{array}{c} V_{0}=\frac{A}{{||A||}_{\infty }^{2}}. \end{array}$$

In fact, by choosing (2), we attain

(3) $$\begin{array}{c} \frac{{||V_{0}||}_{\infty }}{{||A^{-1}||}_{\infty }}=\frac{{||A||}_{\infty }}{{||A||}_{\infty }^{2}{||A^{-1}||}_{\infty }}. \end{array}$$

Based on the fact that *κ*(*A*) = ||*A*||||*A* ^−1^|| ≥ ||*I*|| ≥ 1, we could have

(4) $$\begin{array}{c} \frac{{||V_{0}||}_{\infty }}{{||A^{-1}||}_{\infty }}=\frac{1}{\kappa_{\infty }\left(A\right)}\leq 1, \end{array}$$

which suggests that by choosing (2), the iterative scheme (1) will be almost always convergent for regular matrices.

Some other ways to choose the initial approximation *V*_0_ have been listed in Table 1, where *A* ^*T*^ and A* are the transpose and the conjugate transpose of the complex matrix *A*, respectively, and *N* stands for the size of the square matrix.

A vast discussion on choosing the initial approximation *V*_0_ is given in [6]. For instance, Pan and his coworkers discussed the possible ways to reduce the computational load of Schulz-type iteration methods for structured matrices such as Toeplitz or Hankel matrices; see, for example, [7]. To illustrate further, for a symmetric positive definite (SPD) matrix *A*, one can choose *V*_0_ as follows:

(5) $$\begin{array}{c} V_{0}=\frac{I}{{||A||}_{F}}. \end{array}$$

Another interesting choice is based on [8] for diagonally dominant matrices, which is fruitful when dealing with large scale sparse systems arising from the discretization of differential equations as follows:

(6) $$\begin{array}{c} V_{0}=\mathrm{diag}\left(\frac{1}{a_{11}},\frac{1}{a_{22}},\ldots ,\frac{1}{a_{NN}}\right), \end{array}$$

with *a*_*ii*_ as the diagonal elements of *A*.

Let us now review some of the high order iterative methods for matrix inversion. The perception of the need for higher order methods is the fact that (1) is too slow at the beginning of the process before arriving at the convergence phase for general matrices, and this would increase the computational load of the whole algorithm used for matrix inversion [9].

Li et al. in [10] investigated an iteration of the form

(7) $$\begin{array}{c} V_{n+1}=V_{n}\left(3I-AV_{n}\left(3I-AV_{n}\right)\right),\;\;n=0,1,2,\ldots \end{array}$$

of third-order and also proposed another iterative method for finding *A* ^−1^ of the same order as it is given in

(8) Vn+1=Vn[I+12(I−AVn)(I+(2I−AVn)2)],                   n=0,1,2,….  

Note that a general procedure for constructing such methods was given in ([11], Chapter 5). Krishnamurthy and Sen provided the following fourth-order method:

(9) $$\begin{array}{c} V_{n+1}=V_{n}\left(I+Y_{n}\left(I+Y_{n}\left(I+Y_{n}\right)\right)\right),\;n=0,1,2,\ldots , \end{array}$$

in which *Y*_*n*_ = *I* − *AV*_*n*_. As another example, a ninth-order method could be presented as

(10) Vn+1=Vn(I+Yn(I+Yn(I+Yn(I+Yn(I+Yn(I+Yn(I+Yn(I+Yn)))))))), n=0,1,2,….

In the sequel, we present a new iteration for matrix inversion. Actually, the following section covers our main contribution as a new ninth-order efficient inverse-finding iterative method. We also prove the main theorem therein. Next in Section 3, we discuss the complexity of the iterative methods to theoretically find the most efficient method. In Section 4, we analytically discuss the application of the new algorithm in the computation of the Drazin inverse, which is of interest in numerical analysis.  Section 5 applies the suggested iteration in finding robust approximate inverses for large sparse matrices with real or complex entries in details, while the application of the new scheme in preconditioning of the practical problems will be also given. A clear reduction in the computational time to attain the desired accuracy will be observed therein. Finally, in Section 6 our concluding remarks will be furnished.

## 2. A New Method for Matrix Inversion

A new scheme must be designed by applying an efficient nonlinear (scalar) equation solver to the matrix equation *F*(*V*) = *V* ^−1^ − *A*. Then, one may obtain an iterative process using proper factorization. In this way, by applying the iterative scheme

(11) Yn=Vn−F(Vn)F′(Vn),Zn=Vn−F(Vn)2(1F′(Vn)+1F′(Yn)),Vn+1=Zn−F(Zn)2F′(Zn)(2+F′′(Zn)F(Zn)F′(Zn)2),                  n=0,1,2,…

on the matrix equation *V* ^−1^ − *A* = 0, we are able to find the fixed-point iteration

(12) Vn+1=−18Vn(−7I+9AVn−5(AVn)2+(AVn)3) ×(12I−42AVn+103(AVn)2   −156(AVn)3+157(AVn)4−104(AVn)5   + 43(AVn)6−10(AVn)7+(AVn)8).

Now, by simplification, we suggest the following matrix iteration:

(13) χn=−7I+ψn(9I+ψn(−5I+ψn)),ϑn=ψnχn,Vn+1=−18Vnχn(12I+ϑn(6I+ϑn)),             n=0,1,2,…,

wherein *ψ*_*n*_ = *AV*_*n*_,  *I* is the identity matrix, and the sequence of iterates {*V*_*n*_}_*n*=0_^*n*=*∞*^ converges to *A* ^−1^ under some condition.

In numerical mathematics, it is very useful and essential to know the behavior of an approximate method. Therefore, we are about to prove its order of convergence in Theorem 1.

**Theorem 1**

Let *A* = [*a*_*ij*_] ∈ *ℂ* ^*N*×*N*^ be a nonsingular complex matrix. If the initial approximation *V*_0_ satisfies

(14) $$\begin{array}{c} ||I-AV_{0}||<1, \end{array}$$

then the iterative method (13) converges with ninth order to *A* ^−1^.

**Proof**

We use notations that *E*_0_ = *I* − *AV*_0_ and subsequently *E*_*n*_ = *I* − *AV*_*n*_. Then,

(15)   En+1=I−AVn+1=I−A(−18Vn(−7I+AVn(9I+AVn(−5I+AVn)))     ×(12I+AVn(−7I+AVn(9I+AVn(−5I+AVn)))       ×(6I+AVn(−7I+AVn(9I+AVn(−5I+AVn))))))=I−A(−18Vn(−7I+9AVn−5(AVn)2+(AVn)3)     ×(12I−42(AVn)1+103(AVn)2−156(AVn)3       +157(AVn)4−104(AVn)5+43(AVn)6−10(AVn)7+(AVn)8))=18(−2I+AVn)3(−I+AVn)9=18(I+I−AVn)3(I−AVn)9=18(I+En)3En9=18(En9+3En10+3En11+En12).  

Hence, by taking arbitrary matrix norm on both sides of (15), we attain

(16) $$\begin{array}{c} ||E_{n+1}||\leq \frac{1}{8}\left({||E_{n}||}^{9}+3{||E_{n}||}^{10}+3{||E_{n}||}^{11}+{||E_{n}||}^{12}\right). \end{array}$$

In addition, since ||*E*_0_|| < 1, by relation (16), we obtain that

(17) ||E1||≤18(||E0||9+3||E0||10+3||E0||11+||E0||12)≤||E0||9<1.

Now if we consider ||*E*_*n*_|| < 1, therefore

(18) ||En+1||≤18(||En||9+3||En||10+3||En||11+||En||12)≤||En||9.

Using mathematical induction, we obtain

(19) $$\begin{array}{c} ||E_{n+1}||\leq {||E_{n}||}^{9},\;\;n\geq 0. \end{array}$$

Furthermore, we get that

(20) $$\begin{array}{c} ||E_{n+1}||\leq {||E_{n}||}^{9}\leq \cdots \leq {||E_{0}||}^{9^{n+1}}. \end{array}$$

That is, *I* − *AV*_*n*_ → 0, when *n* → *∞* and

(21) $$\begin{array}{c} V_{n}\to A^{-1},\;\text{as}\;n\to \infty . \end{array}$$

Thus, the new method (13) converges to the inverse of the matrix *A* in the case *ρ*(*AV*_0_) < 1, where *ρ* is the spectral radius. Now, we prove that the order of convergence for the sequence {*V*_*n*_}_*n*=0_^*n*=*∞*^ is at least nine. Let *ε*_*n*_ denotes the error matrix *ε*_*n*_ = *A* ^−1^ − *V*_*n*_; afterwards

(22) $$\begin{array}{c} A\varepsilon_{n}=I-AV_{n}=E_{n}. \end{array}$$

The identity (22) in conjunction with (15) implies that

(23) $$\begin{array}{c} A\varepsilon_{n+1}=\frac{1}{8}\left({\left(A\varepsilon_{n}\right)}^{9}+3{\left(A\varepsilon_{n}\right)}^{10}+3{\left(A\varepsilon_{n}\right)}^{11}+{\left(A\varepsilon_{n}\right)}^{12}\right). \end{array}$$

Therefore, using invertibility of *A*, it follows immediately that

(24) εn+1=18(εn(Aεn)8+3εn(Aεn)9   +3εn(Aεn)10+εn(Aεn)11).

By taking any subordinate norm of (24), we obtain

(25) ||εn+1||≤(18(||A||8+3||A||9||εn||+3||A||10||εn||2   +||A||11||εn||3))||εn||9.

Consequently, it is proved that the iterative formula (13) converges to *A* ^−1^, and the order of this method is at least nine.

The Schulz-type iterations are strongly numerically stable, that is, they have the self-correcting characteristic and are essentially based upon matrix multiplication per an iterative step. Multiplication is effectively parallelizable for structured matrices represented in compressed form.

 The iterative scheme (13) could efficiently be combined with sparse techniques in order to reduce the computational load of matrix-by-matrix multiplications per step. We should also point out that even if the matrix *A* is singular, the Schulz-type methods, including the scheme (13), converge to the Moore-Penrose inverse using a proper initial matrix. A full discussion on this feature of this type of iterative methods has been given in [12].

 Note that {*V*_*n*_}_*n*=0_^*n*=*∞*^ produced from (13), under a certain condition (when *AV*_0_ = *V*_0_ *A*), may be applied not only to the left preconditioned linear system *V*_*n*_ *Ax* = *V*_*n*_ *b* but also to the right preconditioned linear system *AV*_*n*_ *y* = *b*, where *y* = *V*_*n*_ *x*. In fact, an important application of the new method (13) is in preconditioning of the linear system of equations. Practically, experimental results in Section 5 will show that the preconditioner obtained from (13) may lead to nicely clustered eigenvalue distributions of the preconditioned matrices and, hence, results in fast convergence of the preconditioned Krylov subspace iteration methods, such as GMRES and BiCGSTAB for solving some classes of large sparse system of linear equations.

## 3. Complexity of the Methods

 Let us consider the computational complexity of the existing iterative processes (1), (7), (8), (9), (10), and (13), since they are all convergent to *A* ^−1^ in the same condition. From a theoretical analysis, and by assuming a uniform cost for the arithmetic operations, typical of the floating point computations, we consider the *inverse-finder informational efficiency index*. It uses two parameters *ρ* and *κ* which stand for the rate of convergence and the number of matrix-by-matrix multiplications in floating point arithmetics, respectively. Then the comparative index could be expressed by

(26) $$\begin{array}{c} \text{IIEI}=\frac{\rho }{\kappa }. \end{array}$$

Hence, a favorable method in theoretical point of view must reach an order *ρ* with fewer matrix multiplications *κ*, (i.e., *κ* ≤ *ρ*).

 In Table 2, we furnish a comparison on the order along the number of matrix multiplications, rate of convergence, and the index (26) for different methods. The results show that the new established method in Section 2 is better than the others. In fact, by comparing these results, one can see that the iterative process (13) reduces the computational complexity by using less number of basic operations and leads to the better equilibrium between the high speed and the operational cost.

## 4. Application in Finding the Drazin Inverse

In 1958, Drazin in [13] introduced a different kind of generalized inverse in associative rings and semigroups that does not have the reflexivity property but commutes with the element. The importance of this kind of inverse and its computation was later expressed away fully by Wilkinson in [14]. This was the motivation of many authors to develop direct or iterative methods for this important problem; see, for example, [1].

**Definition 2**

The smallest nonnegative integer *k*, such that rank⁡(*A* ^*k*+1^) = rank⁡(*A* ^*k*^), is called the index of *A* and denoted by ind(*A*).

**Definition 3**

Let *A* be an *N* × *N* complex matrix; the Drazin inverse of *A*, denoted by *A* ^*D*^, is the unique matrix *V* satisfying

(27) $$\begin{array}{c} \left(1^{k}\right)A^{k}VA=A^{k},\;\;\left(2\right)VAV=V,\;\;\left(5\right)AV=VA, \end{array}$$

where *k* = ind(*A*) is the index of *A*.

Note that if ind(*A*) = 1, the matrix *V* is called the group inverse of *A*. Also, if *A* is nonsingular, then it is easily seen that ind(*A*) = 0 and *A* ^*D*^ = *A* ^−1^. Note that the idempotent matrix *AA* ^*D*^ is the projector on *ℛ*(*A* ^*k*^) along *𝒩*(*A* ^*k*^), where *ℛ*(*A* ^*k*^) denotes the range of *A* ^*k*^ and *𝒩*(*A* ^*k*^) is the null space of *A* ^*k*^.

 Wei in [15] proved that the general solution of the square singular linear system *Ax* = *b* can be obtained using the Drazin inverse as *x* = *A* ^*D*^ *b* + (*I* − *AA* ^*D*^)*z*, where *z* ∈ *ℛ*(*A* ^*k*−1^) + *𝒩*(*A*).

 In 2004, Li and Wei in [16] proved that the matrix method of Schulz (1) can be used for finding the Drazin inverse of square matrices both possessing real or complex spectra. They proposed the following initial matrix:

(28) $$\begin{array}{c} V_{0}=W_{0}=\alpha A^{l},\;l\geq \text{ind}\left(A\right)=k, \end{array}$$

where the parameter *α* must be chosen so that the condition ||*I* − *AV*_0_|| < 1 is satisfied. Using the initial matrix of the form (28) yields to a matrix method for finding the famous Drazin inverse with quadratical convergence.

 As a consequence, we could present the iterative method of the form (13) with ninth order of convergence for finding the Drazin inverse, where the initial approximation is chosen as

(29) $$\begin{array}{c} V_{0}=W_{0}=\frac{2}{\mathrm{Tr}\left(A^{k+1}\right)}A^{k}, \end{array}$$

wherein *Tr*⁡(·) stands for the trace of an arbitrary square matrix.

 In what follows, we use the following auxiliary results.

**Proposition 4 (see [17])**

Let *M* ∈ *ℂ* ^*N*×*N*^ and *ε* > 0 be given. There is at least one matrix norm ||·|| such that

(30) $$\begin{array}{c} \rho \left(M\right)\leq ||M||\leq \rho \left(M\right)+\epsilon , \end{array}$$

where *ρ*(*M*) denotes the spectral radius of *M*.

**Proposition 5 (see [18])**

If *P*_*L*,*M*_ denotes the projector on a space *L* along a space *M*, then

- *P*_*L*,*M*_ *Q* = *Q* if and only if *ℛ*(*Q*)⊆*L*;
- *QP*_*L*,*M*_ = *Q* if and only if *𝒩*(*Q*)⊇*M*.

**Theorem 6**

Let *A* ∈ *ℂ* ^*N*×*N*^ be singular square matrix. Also, suppose that the initial approximation *W*_0_ is chosen by means of (28). Then the sequence {*W*_*n*_}_*n*=0_^*n*=*∞*^ defined by the iterative method (13) satisfies the following error estimate when finding the Drazin inverse:

(31) $$\begin{array}{c} \frac{||A^{D}-W_{n}||}{||A^{D}||}\leq {||I-AW_{0}||}^{9^{n}}. \end{array}$$

**Proof**

Consider the notation *F*_0_ = *I* − *AW*_0_ and subsequently the residual matrix as *F*_*n*_ = *I* − *AW*_*n*_. Then similarly as in (15), we get

(32) Fn+1=I−AWn+1=I−A(−18Wn(−7I+AWn(9I+AWn(−5I+AWn)))     ×(12I+AWn(−7I+AWn(9I+AWn(−5I+AWn)))       ×(6I+AWn(−7I+AWn(9I+AWn(−5I+AWn))))))=18(I+I−AWn)3(I−AWn)9=18(Fn9+3Fn10+3Fn11+Fn12).

By taking an arbitrary matrix norm on both sides of (32), we attain

(33) $$\begin{array}{c} ||F_{n+1}||\leq \frac{1}{8}\left({||F_{n}||}^{9}+3{||F_{n}||}^{10}+3{||F_{n}||}^{11}+{||F_{n}||}^{12}\right). \end{array}$$

In addition, since ||*F*_0_|| < 1, by relation (33), we obtain that ||*F*_1_|| ≤ (1/8)(||*F*_0_||^9^ + 3||*F*_0_||^10^ + 3||*F*_0_||^11^ + ||*F*_0_||^12^) ≤ ||*F*_0_||^9^. Similarly, ||*F*_*n*+1_|| ≤ (1/8)(||*F*_*n*_||^9^ + 3||*F*_*n*_||^10^ + 3||*F*_*n*_||^11^ + ||*F*_*n*_||^12^) ≤ ||*F*_*n*_||^9^. Using mathematical induction, we obtain ||*F*_*n*+1_|| ≤ ||*F*_*n*_||^9^ for each *n* ≥ 0. This implies that

(34) $$\begin{array}{c} {||F_{n}||}^{9}\leq {||F_{0}||}^{9^{n}},\;n\geq 0. \end{array}$$

Since *W*_0_ is chosen as in (28), *ℛ*(*W*_0_)⊆*ℛ*(*A* ^*k*^) immediately follows. Further, using this fact in conjunction with (12), which implies that *ℛ*(*W*_*n*_)⊆*ℛ*(*W*_*n*−1_), we conclude that

(35) $$\begin{array}{c} ℛ\left(W_{n}\right)\subseteq ℛ\left(A^{k}\right),\;n\geq 0. \end{array}$$

Similarly, if we rewrite (13) in the form

(36) Wn+1=−18(−7I+9WnA−5(WnA)2+(WnA)3) ×(12I−42AWn+103(WnA)2   −156(WnA)3+157(WnA)4   −104(WnA)5+43(WnA)6   −10(WnA)7+(WnA)8)Wn.

it is not difficult to verify that

(37) $$\begin{array}{c} 𝒩\left(W_{n}\right)⊇𝒩\left(A^{k}\right),\;n\geq 0. \end{array}$$

Now, an application of the well-known results from [12]

(38) $$\begin{array}{c} AA^{D}=A^{D}A=P_{ℛ(A^{k}),𝒩(A^{k})} \end{array}$$

in conjunction with Proposition 5 and (35), (37), immediately follows

(39) $$\begin{array}{c} W_{n}AA^{D}=W_{n}=A^{D}AW_{n},\;n\geq 0. \end{array}$$

Therefore, the error matrix *δ*_*n*_ = *A* ^*D*^ − *W*_*n*_ satisfies

(40) $$\begin{array}{c} \delta_{n}=A^{D}-W_{n}=A^{D}-A^{D}AW_{n}=A^{D}\left(I-AW_{n}\right)=A^{D}F_{n}. \end{array}$$

From the last identity and (34) we have

(41) $$\begin{array}{c} ||\delta_{n}||=||A^{D}||||F_{n}||\leq ||A^{D}||{||F_{0}||}^{9^{n}}, \end{array}$$

which is a confirmation of (31).

The following result is a consequence of Theorem 6.

**Corollary 7**

If the conditions of Theorem 6 are satisfied and the initial iteration *W*_0_ is chosen such that

(42) $$\begin{array}{c} ||F_{0}||=||I-AW_{0}||<1, \end{array}$$

the iterative method (13) converges to *A* ^*D*^.

Therefore, our goal is to find initial approximations *W*_0_ satisfying (42). In accordance with Proposition 4, *W*_0_ must satisfy the following inequality to ensure the convergence in the Drazin inverse case:

(43) $$\begin{array}{c} \underset{1\leq i\leq t}{\max}\left|1-\lambda_{i}\left(AW_{0}\right)\right|<1, \end{array}$$

where rank⁡(*AW*_0_) = *t* and *λ*_*i*_(*AW*_0_), *i* = 1,…, *t* are eigenvalues of *AW*_0_.

**Theorem 8**

Let *A* ∈ *ℂ* ^*N*×*N*^ be a singular square matrix with ind (*A*) = *k*, and the sequences *F*_*n*_, *δ*_*n*_ are defined as in Theorem 6. The sequence {*W*_*n*_}_*n*=0_^*n*=*∞*^ defined by the iterative method (13) converges with ninth order to *A* ^*D*^ if the initial approximation *W*_0_ is in accordance with (42).

**Proof**

Now, by considering *δ*_*n*_ = *A* ^*D*^ − *W*_*n*_ as the error matrix for finding the Drazin inverse, we have

(44) Aδn+1=AAD−AWn+1=AAD−I+I−AWn+1=AAD−I+Fn+1.

Taking into account (32) and using elementary algebraic transformations, we further derive

(45) Aδn+1=AAD−I+18(Fn9+3Fn10+3Fn11+Fn12)=18((Fn9+3Fn10+3Fn11+Fn12)+8(AAD−I))=18((Fn9+AAD−I)+3(Fn10+AAD−I)   + 3(Fn11+AAD−I)+(Fn12+AAD−I)).

Now, using the idempotent property (*I* − *AA* ^*D*^)^*t*^ = (*I* − *AA* ^*D*^), *t* ≥ 1, and the following consequence of (39)

(46) (I−AAD)Aδn=(I−AAD)A(AD−Wn)=Wn−AADWn=0,

we obtain for each *t* ≥ 1 the following:

(47) (Fn)t+AAD−I=(I−AWn)t+AAD−I=(I−AAD+AAD−AWn)t+AAD−I=((I−AAD)+Aδn)t+AAD−I=I−AAD+(Aδn)t+AAD−I=(Aδn)t.

From (47) and (45),

(48) $$\begin{array}{c} A\delta_{n+1}=\frac{1}{8}\left({\left(A\delta_{n}\right)}^{9}+3{\left(A\delta_{n}\right)}^{10}+3{\left(A\delta_{n}\right)}^{11}+{\left(A\delta_{n}\right)}^{12}\right) \end{array}$$

Therefore,

(49) ||Aδn+1||≤18(||Aδn||9+3||Aδn||10+3||Aδn||11+||Aδn||12)≤||Aδn||9.

Finally, applying (39), it is now easy to find the error inequality of the new scheme (13) using (49) and the second condition of (27), when finding the Drazin inverse, as follows:

(50) ||δn+1||=||Wn+1−AD||=||ADAWn+1−ADAAD||=||AD(AWn+1−AAD)||≤||AD||||Aδn+1||≤||AD||||A||9||δn||9.

Therefore, since (42) is satisfied, from (31) follows *δ*_*n*_ → 0. Furthermore, the inequalities in (50) immediately lead to the conclusion that *W*_*n*_ → *A* ^*D*^ as *n* → +*∞* with the ninth order of convergence.

## 5. Numerical Aspects

Using the programming package Mathematica 8 [19] in this section, we apply our iterative method on some practical numerical tests and compare it with the existing methods in order to manifest the applicability and the consistency of numerical results with the theoretical aspects illustrated in Sections 2–4.

For numerical comparisons in this section, we have used the methods (1), (7), (8), (9), (10), and (13) denoted by “Schulz”, “Li et al. I”, “Li et al. II”, “KMS4”, “KMS9,” and the “Proposed method”, respectively. We have carried out the numerical tests with machine precision on a computer with Pentium 4. In fact, the computer characteristics are Microsoft Windows XP Intel(R), Pentium(R) 4 CPU, 3.20 GHz with 4 GB of RAM. In all computations, the running time in seconds using AbsoluteTiming[] was attained.

In sparse-matrix algebra, the iteration methods such as (1), (7), (8), (9), (10) and (13) should be coded in sparse form using some well-known commands such as SparseArray[] to reduce the computational burden and preserve the sparsity feature of the approximate inverse per computing step. This is done herein along with a threshold by applying Chop[] on each approximate inverse *V*_*i*_.

Now, we apply the above inverse finders for finding the approximate inverses of the following large sparse matrices.

**Test Problem 1**

In this test, 10 large sparse random complex matrices of the dimension 5000 are considered as shown in Algorithm 1.

Note that $I=\sqrt{-1}$. In this test, the stopping criterion is ||*V*_*n*+1_−*V*_*n*_||_1_ ≤ 10^−6^ and the maximum number of iterations allowed is set to 75. Note that in this test the initial choice has been constructed using different available ways as discussed in Table 1.

**Figure 1:**
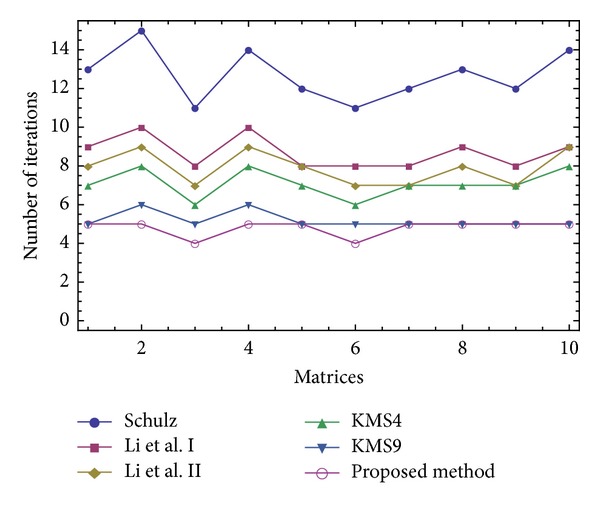
Comparison of the number of evaluations for solving the Test Problem 1 using *V*_0_ = *A*/||*A*||_1_^2^.

**Figure 2:**
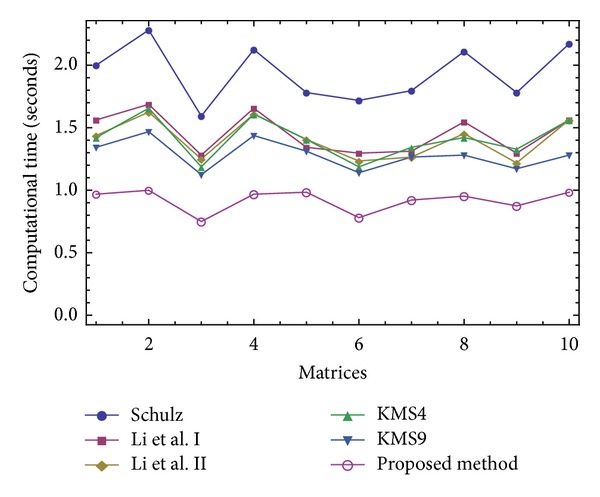
Comparison of the elapsed time for solving the Test Problem 1 using *V*_0_ = *A*/||*A*||_1_^2^.

**Figure 3:**
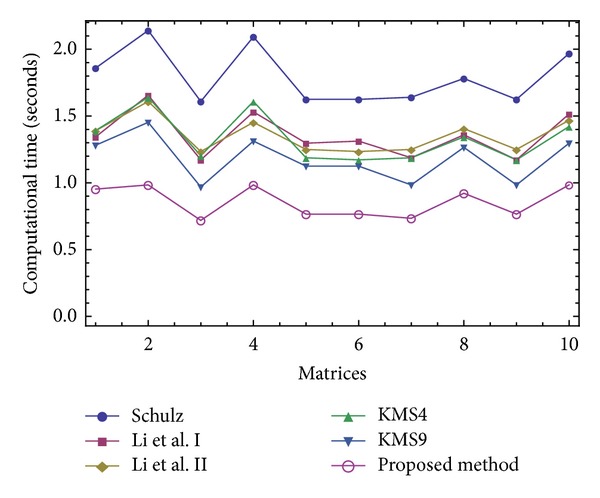
Comparison of the elapsed time for solving the Test Problem 1 using *V*_0_ = *A*/||*A*||_*∞*_^2^.

**Figure 4:**
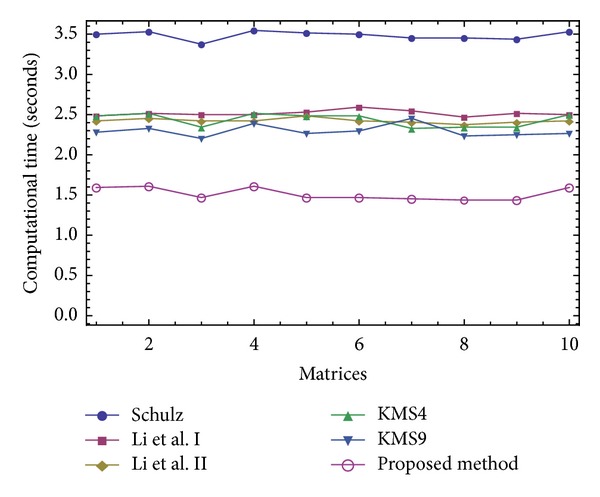
Comparison of the elapsed time for solving the Test Problem 1 using *V*_0_ = *A*/||*A*||_*F*_^2^.

 The results of comparisons for this test have been presented in Figures 1, 2, 3, and 4. In Figure 1, we show the number of iterations required by different methods to attain the desired accuracy when *V*_0_ = *A*/||*A*||_1_^2^. As it is obvious that higher order methods require lower number of iterations to converge, we then put our focus on the computational time needed to satisfy the desired tolerance using three different initial approximations *V*_0_ = *A*/||*A*||_1_^2^, *V*_0_ = *A*/||*A*||_*∞*_^2^, and *V*_0_ = *A*/||*A*||_*F*_^2^. As could be observed for all the three forms of the initial guesses and in all the 10 test problems (given in Figures 2–4), our iterative method (13) beats the other existing schemes, which immediately follows the theoretical results of Table 2.

The attained results have reverified the robustness of the proposed iterative method (13) by a *clear reduction* in the number of iterations and *the elapsed time*.

The practical application of the new scheme (13) falls within many problems as discussed in Section 1. For instance, in solving second-kind integral equations by Wavelet-like approach, the problem will be reduced to find that the inverse of a large sparse matrix possesses a sparse inverse as well [20]. In the rest of this section, we apply our new iterative method as a robust technique to produce accurate preconditioners for accelerating modern iterative solvers such as GMRES or BiCGSTAB for solving large scale sparse linear systems; see, for example, [21].

**Test Problem 2**

Consider solving the following Boundary Value Problem (BVP) using discretization. In such problems, and in order to capture all the shocks and the behavior of the solution, a very fine grid of points is required in *𝔽𝔻* approach:

(51) $$\begin{array}{c} y^{\prime \prime }=3y-2y^{\prime },\;\;t\in \left[a,b\right],\\ y\left(a\right)=e^{3},\\ y\left(b\right)=e^{-3}, \end{array}$$

where the domain of the solution is *a* = 0 and *b* = 2. To solve (51) using 3-point *𝔽𝔻* discretization, we assume that in the grid points *t*_*i*_, the exact solution is denoted by *y*(*t*_*i*_) and the approximate solution is defined by *w*_*i*_, for any *i* = 0,1,…, *n*, *n* + 1.

Thus, (51) can be written in discretized form in Mathematica language (see Algorithm 2), wherein *n* is the number of grid points which result in an *n* × *n* sparse linear system of equations. In this test, we consider the tolerance as 10^−8^ for the final solution of the linear systems. The results of solving this system “without preconditioning” and “with left preconditioning” are given in Tables 3 and 4. Note that in Tables 3 and 4, for example, PBiCGSTAB-(8)-II stands for the left preconditioned linear system (*V* *Ax* = *Vb*) applying the preconditioner (approximative inverse) obtained by the scheme (8) after 2 iterations, while it is solved by the iterative solver BiCGSTAB.

 The numerical results clearly support the efficiency of the method (13). A clear reduction in the *elapsed time* is observable. Even for the case of GMRES solver which had failed (not convergent after 1500 iterations to the considered tolerance), a simple preconditioner obtained by the new method (13) significantly improved the problem. Note that in this test, we have constructed *V*_0_ for the compared methods using (6).

**Test Problem 3**

The aim of this example is to apply the discussions of Section 4, for finding the Drazin inverse of the following square matrix (taken from [16]):

(52) $$\begin{array}{c} A=\left[\begin{array}{cccccccccccc} 2 & 0.4 & 0 & 0 & 0 & 0 & 0 & 0 & 0 & 0 & 0 & 0\\ -2 & 0.4 & 0 & 0 & 0 & 0 & 0 & 0 & 0 & 0 & 0 & 0\\ -1 & -1 & 1 & -1 & 0 & 0 & 0 & 0 & -1 & 0 & 0 & 0\\ -1 & -1 & -1 & 1 & 0 & 0 & 0 & 0 & 0 & 0 & 0 & 0\\ 0 & 0 & 0 & 0 & 1 & 1 & -1 & -1 & 0 & 0 & -1 & 0\\ 0 & 0 & 0 & 0 & 1 & 1 & -1 & -1 & 0 & 0 & 0 & 0\\ 0 & 0 & 0 & -1 & -2 & 0.4 & 0 & 0 & 0 & 0 & 0 & 0\\ 0 & 0 & 0 & 0 & 2 & 0.4 & 0 & 0 & 0 & 0 & 0 & 0\\ 0 & -1 & 0 & 0 & 0 & 0 & 0 & 0 & 1 & -1 & -1 & -1\\ 0 & 0 & 0 & 0 & 0 & 0 & 0 & 0 & -1 & 1 & -1 & -1\\ 0 & 0 & 0 & 0 & 0 & 0 & 0 & 0 & 0 & 0 & 0.4 & -2\\ 0 & 0 & 0 & 0 & 0 & 0 & 0 & 0 & 0 & 0 & 0.4 & 2 \end{array}\right], \end{array}$$

with *k* = ind(*A*) = 3. To simplify the process, we write a general code in the programming package Mathematica for the iterative process (13), to find the (pseudo-)inverse or the Drazin inverse of arbitrary matrices (see Algorithm 3).

The two-argument function DrazinInverse[A_,tolerance_] takes the arbitrary matrix *A* and the tolerance from the user to obtain its Drazin inverse by knowing the index *k*. In this case, by choosing tolerance = 10^−8^, we obtain

(53) $$\begin{array}{c} A^{D}=\left[\begin{array}{cccccccccccc} 0.25 & -0.25 & 0. & 0. & 0. & 0. & 0. & 0. & 0. & 0. & 0. & 0.\\ 1.25 & 1.25 & 0. & 0. & 0. & 0. & 0. & 0. & 0. & 0. & 0. & 0.\\ -1.66406 & -0.992187 & 0.25 & -0.25 & 0. & 0. & 0. & 0. & -0.0625 & -0.0625 & 0. & 0.15625\\ -1.19531 & -0.679687 & -0.25 & 0.25 & 0. & 0. & 0. & 0. & -0.0625 & 0.1875 & 0.6875 & 1.34375\\ -2.76367 & -1.04492 & -1.875 & -1.25 & -1.25 & 1.25 & 1.25 & 1.25 & 1.48438 & 2.57813 & 3.32031 & 6.64063\\ -2.76367 & -1.04492 & -1.875 & -1.25 & -1.25 & 1.25 & 1.25 & 1.25 & 1.48438 & 2.57813 & 4.57031 & 8.51563\\ 14.1094 & 6.30078 & 6.625 & 3.375 & 5. & -3. & -5. & -5. & -4.1875 & -8.5 & -10.5078 & -22.4609\\ -19.3242 & -8.50781 & -9.75 & -5.25 & -7.5 & 4.5 & 7.5 & 7.5 & 6.375 & 12.5625 & 15.9766 & 33.7891\\ -0.625 & -0.3125 & 0. & 0. & 0. & 0. & 0. & 0. & 0.25 & -0.25 & -0.875 & -1.625\\ -1.25 & -0.9375 & 0. & 0. & 0. & 0. & 0. & 0. & -0.25 & 0.25 & -0.875 & -1.625\\ 0. & 0. & 0. & 0. & 0. & 0. & 0. & 0. & 0. & 0. & 1.25 & 1.25\\ 0. & 0. & 0. & 0. & 0. & 0. & 0. & 0. & 0. & 0. & -0.25 & 0.25 \end{array}\right]. \end{array}$$

Checking the conditions of Definition 3 yields to

(54) $$\begin{array}{c} {||A^{k+1}A^{D}-A^{k}||}_{\infty }=1.48415\;\times \;10^{-12},\\ {||A^{D}AA^{D}-A^{D}||}_{\infty }=1.20264\;\times \;10^{-10},\\ {||AA^{D}-A^{D}A||}_{\infty }=8.93836\;\times \;10^{-11}, \end{array}$$

which supports the theoretical discussions.

## 6. Conclusion

 In many engineering applications, extracting the diagonal or the whole entries of the inverse of a given matrix (basically large and sparse) is an important part of the computation, for example, in electronic structure calculation and especially for models based on effective one-electron Hamiltonians such as the tight-binding models, or in modern statistics, and thus, developing high-order efficient Schulz-type methods is of practical interest.

In this paper, we have studied a high-order iteration method (13) for matrix inversion. Convergence analysis of our iterative algorithm has been studied and a discussion on the choice of the initial value in order to start the process and preserve the convergence order has been given. We also discussed that under what conditions a new method could be applied for finding the Drazin inverse of square matrices having real or complex spectra.

As a result, the total time consuming of the suggested iteration (13) is remarkably low in contrast with the existing methods of this type in the case of constructing approximate inverses and in preconditioning.

Working on the extension of the proposed method (13) for interval matrix inversion [22] can be considered as future works in this field of study.

## Figures and Tables

**Algorithm 1:**
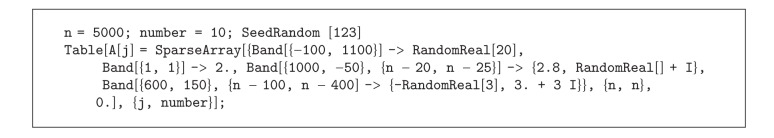


**Algorithm 2:**
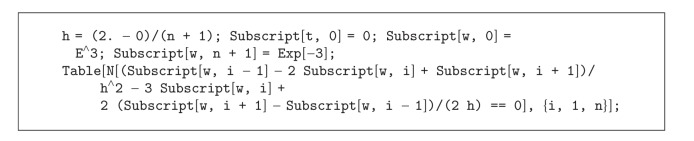


**Algorithm 3:**
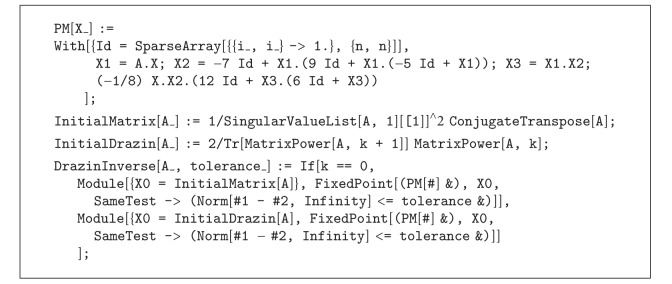


**Table 1:** Some of the general ways to choose *V*_0_.

Forms	Form 1	Form 2	Form 3	Form 4	Form 5
Initial formulation	*A*/||*A*||_1_^2^	*A*/||*A*||_∞_^2^	*A*/||*A*||_*F*_^2^	*A*^*T*/(*N*||*A*||_1_||*A*||_∞_ )	*A*^∗/||*A*||_2_^2^

**Table 2:** Comparison of the computational complexity for different methods.

Methods	(1)	(7)	(8)	(9)	(10)	(13)
Rate of convergence	2	3	3	4	9	9
Number of matrix multiplications	2	3	4	4	9	7
IIEI	2/2 = 1	3/3 = 1	3/4 = 0.75	4/4 = 1	9/9 = 1	9/7 ≈ 1.285

**Table 3:** Comparison of the computational time in solving the linear system resulting of discretization of (51) when *n* = 1500.

Methods	GMRES	PGMRES-(1)-VI	PGMRES-(7)-IV	PGMRES-(8)-III	PGMRES-(9)-III	PGMRES-(13)-II
Total time	Fail	3.50	1.78	1.53	2.62	1.31

**Table 4:** Comparison of the computational time in solving the linear system resulting of discretization of (51) when *n* = 2000.

Methods	BiCGSTAB	PBiCGSTAB-(1)-III	PBiCGSTAB-(7)-III	PBiCGSTAB-(8)-II	PBiCGSTAB-(9)-II	PBiCGSTAB-(13)-I
Total time	1.98	0.53	0.60	0.53	0.54	0.46
